# Subject‐Level Segmentation Precision Weights for Volumetric Studies Involving Label Fusion

**DOI:** 10.1002/hbm.70082

**Published:** 2024-12-19

**Authors:** Christina E. Chen, Sandhitsu R. Das, M. Dylan Tisdall, Fengling Hu, Andrew A. Chen, Paul A. Yushkevich, David A. Wolk, Russell T. Shinohara, for the Alzheimer's Disease Neuroimaging Initiative

**Affiliations:** ^1^ Penn Statistics in Imaging and Visualization Endeavor (PennSIVE), Department of Biostatistics, Epidemiology, and Informatics Perelman School of Medicine, University of Pennsylvania Philadelphia Pennsylvania USA; ^2^ Penn Image Computing and Science Laboratory (PICSL), Department of Radiology University of Pennsylvania Philadelphia Pennsylvania USA; ^3^ Center for Biomedical Image Computing and Analytics (CBICA), Department of Radiology University of Pennsylvania Philadelphia Pennsylvania USA; ^4^ Department of Radiology University of Pennsylvania Philadelphia Pennsylvania USA; ^5^ Department of Public Health Sciences Medical University of South Carolina Charleston South Carolina USA; ^6^ Penn Memory Center, Department of Neurology University of Pennsylvania Philadelphia Pennsylvania USA

## Abstract

In neuroimaging research, volumetric data contribute valuable information for understanding brain changes during both healthy aging and pathological processes. Extracting these measures from images requires segmenting the regions of interest (ROIs), and many popular methods accomplish this by fusing labels from multiple expert‐segmented images called atlases. However, post‐segmentation, current practices typically treat each subject's measurement equally without incorporating any information about variation in their segmentation precision. This naïve approach hinders comparing ROI volumes between different samples to identify associations between tissue volume and disease or phenotype. We propose a novel method that estimates the variance of the measured ROI volume for each subject due to the multi‐atlas segmentation procedure. We demonstrate in real data that weighting by these estimates markedly improves the power to detect a mean difference in hippocampal volume between controls and subjects with mild cognitive impairment or Alzheimer's disease.

## Graphical abstract

We propose a novel method that estimates subject‐level precision weights for ROI volume estimates produced by multi‐atlas segmentation. We demonstrate in real data that incorporating these weights markedly improves the power to detect a mean difference in hippocampal volume between controls and subjects with mild cognitive impairment or Alzheimer's disease.

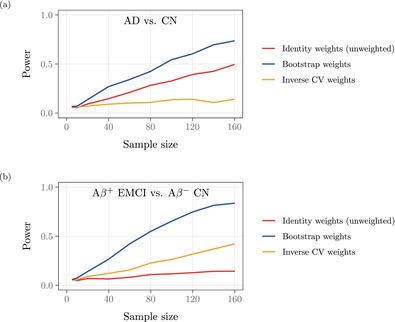

## 1 Introduction

Region‐specific changes in brain volume accompany many neurological and neuropsychiatric diseases. Detecting and delineating these changes can link structural and clinical findings to illuminate disease biology and facilitate clinical management. For example, Alzheimer's disease (AD) is a neurodegenerative disorder characterized by progressive cognitive decline, a feature shared by other diseases such as dementia with Lewy bodies (McKeith et al. 2017). Its definite diagnosis requires autopsy findings of neuropathological deposits, which concentrate in characteristic regions such as the hippocampus, a brain structure associated with learning and memory (DeTure and Dickson 2019; Serrano‐Pozo et al. 2011). Magnetic resonance imaging (MRI) volumetric studies have complemented and augmented these findings by establishing correlations between the magnitude and rate of hippocampal atrophy and the severity and progression of clinical disease (Jack Jr. et al. 2011).

Comparing hippocampal volumes among cognitively normal (CN) controls and subjects with mild cognitive impairment (MCI) or AD in a neuroimaging study entails first segmenting the hippocampus in each study image. Manual segmentation is cumbersome and susceptible to errors and inter‐observer variability. Automated atlas‐based segmentation circumvents some of these shortcomings by borrowing information from expert‐labeled images (called atlases) to segment target images.

Single‐atlas segmentation comprises registering an atlas to the target image and warping the atlas labels via the deformation determined by the registration algorithm to obtain a segmentation of the target image (Iglesias and Sabuncu 2015; Rohlfing et al. 2005). Multi‐atlas label fusion (MALF) refines single‐atlas segmentation by aggregating the labels from multiple atlases. Intuitively, reconciling the decisions of multiple atlases dilutes the mistakes of each single atlas. Early proponents of MALF (Heckemann et al. 2006; Klein et al. 2005; Rohlfing et al. 2004) referenced observations from pattern recognition that combining several independent classifiers improves classification accuracy. Voxel‐wise majority voting, which labels each voxel by the label endorsed by the most number of atlases, is a simple example of MALF. Most existing MALF methods implement some version of voting that weights atlases (Iglesias and Sabuncu 2015). For example, global weighting assigns a global weight to each atlas according to its overall alignment with the target image. In contrast, local weighting, which accommodates the spatial heterogeneity of each atlas's registration accuracy, assigns local (region‐wise or voxel‐wise) weights to each atlas according to its alignment with the target image within different regions or voxel neighborhoods.

One straightforward way of testing for a difference in hippocampal volume between cases and controls involves segmenting the target images to estimate the hippocampal volume for each subject and then conducting a two‐sample t‐test or regressing the volume estimates against the group labels. These approaches equalize the contributions of all study subjects. Unfortunately, image degradation by noise or motion artifacts hinders segmentation, and segmentations that misidentify regional boundaries can output inaccurate volume estimates that attenuate power or even induce bias (Alexander‐Bloch et al. 2016; Reuter et al. 2015). However, discarding poorly segmented images altogether requires imposing an absolute threshold on segmentation quality and can potentially deplete the sample size. Ideally, we would prefer weighting the volume estimate from each target image according to its precision.

We propose a novel, statistically motivated method of deriving subject‐level precision weights by quantifying how resampling different sets of atlases for joint segmentation perturbs each subject's volume estimate. This approach reconciles theory with our intuition about image behavior during registration and the attribution of most segmentation inaccuracy to registration errors (Wang et al. 2013). Since most commonly used registration algorithms employ stochastic optimization, a target image that deviates significantly from the atlas (due to biological factors, motion artifacts, etc.) will induce the optimization process to arbitrarily locate one of several similarly uninformative local optima. Consequently, the transformed labels from different atlases will differ more compared to those of a better‐aligned target image that admits a sharper optimum, and these variations will propagate into joint segmentations produced by different atlas collections. Thus, for a fixed target image, large variability across volume estimates from our resampling procedure indicates low precision in the segmentation that produced our original estimate.

In Section 2, we describe the subsets of images from the Alzheimer's Disease Neuroimaging Initiative (ADNI) that we used to validate our proposed precision weights. In Section 3, we mathematically formalize the method that we described above and specify how to estimate subject‐level weights. We also describe the experiments that we conducted to compare our weights to previously published MRI quality metrics and to evaluate how incorporating these weights influences power and type I error rate in detecting a mean difference in hippocampal volume between ADNI subgroups. In Section 4, we compile our results. In Section 5, we conclude by discussing generalizations and extensions of our method.

## 2 Data

Data used in the preparation of this article were obtained from the ADNI database (adni.loni.usc.edu). As such, the investigators within the ADNI contributed to the design and implementation of ADNI or provided data but did not participate in the analysis or writing of this report.

The ADNI was launched in 2003 as a public–private partnership led by Principal Investigator Michael W. Weiner, MD. The primary goal of ADNI has been to test whether serial MRI, positron emission tomography (PET), other biological markers, and clinical and neuropsychological assessment can be combined to measure the progression of MCI and early AD. It is a longitudinal, multicenter study that has recruited both healthy controls and subjects with MCI and AD from multiple sites across the United States (Petersen et al. 2010).

We used T1‐weighted MRIs acquired during the ADNI 2 and ADNI 3 phases of the ADNI study. For our experiment comparing controls (CN) to subjects with AD, we used images from 200 controls and 200 AD subjects. The control group comprises 94 males and 106 females, with ages in the range of 55.2–94.7 (mean 74.7). The AD group comprises 120 males and 80 females, with ages in the range of 55.3–90.4 (mean 74.8). For our experiment comparing controls to subjects with early MCI (EMCI), we used images from 200 amyloid‐negative controls and 200 amyloid‐positive EMCI subjects. We determined the amyloid‐β status by thresholding the composite SUVR scores provided by the ADNI (≥1.11 and ≥1.08 for 18F‐florbetapir and 18F‐florbetaben amyloid PET scans, respectively, as described in Landau et al. (2013)). The amyloid‐negative control group comprises 104 males and 96 females, with ages in the range of 55.2–94.7 (mean 72.9). The amyloid‐positive EMCI group comprises 115 males and 85 females, with ages in the range of 55.5–89.0 (mean 74.2).

Amyloid‐β deposition in the brain constitutes a hallmark feature of AD, and PET imaging can detect abnormal amyloid burden in AD patients years before they manifest significant cognitive decline. This biomarker typically precedes the detection of hippocampal atrophy by structural MRI (Bjorkli, Sandvig, and Sandvig 2020). Thus, we constructed our second experiment as a more difficult test to differentiate between healthy controls and subjects with mild memory impairment and AD pathology, who will likely eventually progress to AD but might not yet have sustained sufficiently severe hippocampal neuronal loss to generate MRI findings.

We neck‐trimmed our ADNI T1‐weighted images before performing N4 bias correction (Tustison et al. 2010) using the Advanced Normalization Tools package as implemented in R (ANTsR) and skull‐stripping using the brain extraction tool (bet) in the FMRIB Software Library (FSL) (Smith 2002).

For multi‐atlas segmentation, we used the 35 OASIS (Marcus et al. 2010) atlases provided by Landman and Warfield (2012) and used to validate the popular MUSE segmentation method proposed by Doshi et al. (2016). The atlases were acquired from 30 healthy volunteers (20 females, 10 males), five of whom contributed two scans. The ages of the atlas subjects were in the range 18–90 (mean 34.3). The hippocampal volumes in these atlases were in the range 5.7–9.2 (mean 7.4) cm^3^.

We registered the 35 OASIS atlases to the processed ADNI images using the symmetric normalization (SyN) algorithm (Avants et al. 2008) implemented in ANTsR. We fused the labels from the registered atlases via joint label fusion (JLF), a widely used multi‐atlas label fusion method (Wang et al. 2013). JLF implements weighted voting and computes voxel‐specific weights by leveraging the insight that different atlases incur correlated errors depending on a voxel's inherent labeling difficulty. In our case, we employed JLF to obtain whole brain segmentations to extract the hippocampus, but JLF also constitutes the primary fusion step for algorithms such as Automated Segmentation of Hippocampal Subfields (ASHS, Yushkevich et al. 2014) that segment the hippocampal subfields.

## 3 Methods

### 3.1 Precision Weights: Theory

Consider a study comprising target images from n subjects. Let $T_{i}$ denote the target image for the i‐th subject. Suppose that we segment $T_{i}$ via multi‐atlas segmentation using atlases $A_{1},\ldots ,A_{m}$ to derive a hippocampal volume estimate $Y_{i}\in \mathbb{R}$. Let $X_{i}\in {\mathbb{R}}^{p}$ denote a vector of covariates for the i‐th subject (i.e. age, disease status, intracranial brain volume, etc.) Assume the data‐generating model

$$Y_{i}=X_{i}^{t}\beta +\epsilon_{i},$$

where $\beta \in {\mathbb{R}}^{p}$ denotes the fixed covariate effects and $\epsilon_{i}$ denotes the random error term associated with $Y_{i}$. Assume that $\epsilon_{i}$ is independent of $T_{j}$ for i≠j. Also, assume that the errors have expectation 0 and
(1)

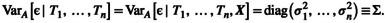




That is, the error terms are uncorrelated given the target images and knowing the covariates $X\in {\mathbb{R}}^{n\times p}$ does not diminish segmentation uncertainty when the target images are available. We include the subscript in (1) to emphasize that the atlases comprise the source of randomness but will omit it hereafter for conciseness.

We may estimate β via multivariable ordinary least‐squares (OLS) regression, which minimizes the sum of the squared residuals. In the presence of heteroskedasticity (i.e., the $Y_{i}$ do not share the same variance), we can calibrate our estimate by minimizing a weighted sum of the squared residuals. The weighted least‐squares estimator (WLS) determined by the weight matrix $W\equiv \mathrm{diag}\left(w_{1},\ldots ,w_{n}\right)$ admits the closed‐form formula ${\left(X^{t}\mathrm{WX}\right)}^{-1}X^{t}\mathrm{WY}$ and is an unbiased estimator of β. It is well‐known that inverse variance‐weighting minimizes the variances of the estimated coefficients in the case of uncorrelated errors (Aitken 1935). (We include the formal proposition and proof in the supplement.) That is, if we assume that the volume estimates are independently conditioned on the target images, then weighting each $Y_{i}$ by $\mathrm{Var}\left[Y_{i}\;|\;T_{i}\right]$ minimizes the conditional variance $\mathrm{Var}\left[\hat{\beta }\;|\;T_{1},\ldots ,T_{n}\right]$ of the estimated group effect size $\hat{\beta }$. Thus, in this case, the theory establishes that the optimal weight for $Y_{i}$ equals $\mathrm{Var}{\left[Y_{i}\;|\;T_{i}\right]}^{-1}$, its precision (inverse variance).

Our desired variances $\mathrm{Var}{\left[Y_{i}\;|\;T_{i}\right]}^{-1}$ describe a framework that fixes the target image while varying the atlases. The complexity of the space of atlases precludes invoking any existing theoretical approach, so we elected to estimate these variances by bootstrapping on the atlases. Bootstrapping is a common and versatile technique for calibrating the properties of a sample estimate (Efron 1979). If our atlas collection encapsulates the space of all reasonable atlases, then spawning new atlas collections by sampling with replacement from our original atlas collection simulates sampling multiple atlas collections from the entire space of atlases. Thus, for each target image, the distribution of volume estimates computed from the resampled (called bootstrapped) atlas collections resembles its sampling distribution. In particular, the variance of these volume estimates approximates the variance of our original volume estimate.

### 3.2 Precision Weights: Computation

**FIGURE 1:**
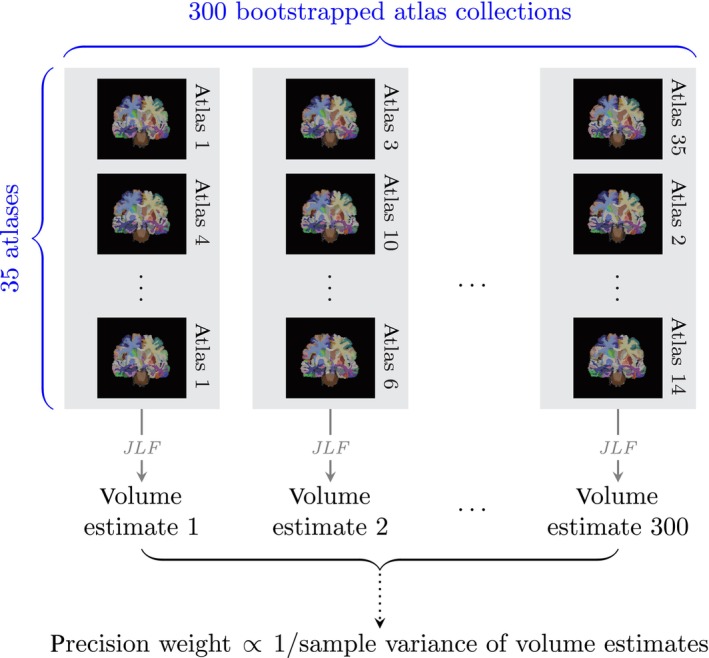
For each subject, we obtained 300 bootstrapped atlas collections (each constructed by sampling with replacement from the 35 OASIS atlases), extracted the hippocampal volume from the JLF segmentation produced by each atlas collection, computed the sample variance across these 300 volume estimates, and inverted this variance to assign a subject‐specific precision weight. This schematic emphasizes the facility of computing a precision weight for any joint segmentation‐derived volume estimate, regardless of the MALF method used. After obtaining our bootstrapped atlas collections, applying the corresponding MALF method to each collection produces the volume estimates required to estimate the relevant variance.

For each target image, we (1) obtained 300 bootstrapped atlas collections (of size 35) by sampling with replacement from our 35 OASIS atlases, (2) derived the hippocampal volume from the whole brain segmentation produced by fusing the labels from each atlas collection, and (3) computed the variance across these 300 volume estimates. As described in Section 3.1, we inverted this variance to weight the hippocampal volume estimate for each subject. See Figure 1 for a schematic illustrating our estimation procedure for each target image.

**FIGURE 2:**
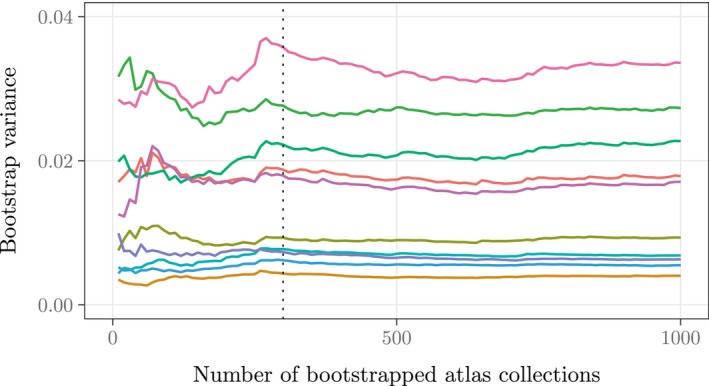
For each of the first 10 control subjects in our ADNI subset, we obtained 1000 bootstrapped atlas collections (each constructed by sampling with replacement from the 35 OASIS atlases) and extracted the hippocampal volume from the JLF segmentation produced by each atlas collection. Each colored line tracks the sample variance of a particular subject's first n bootstrapped volume estimates (in cm^3^) as n ranges from 10 to 1000. The dotted line at n=300 indicates the bootstrap size that we selected for our experiments.

We determined the number of bootstrap replicates required to approximate the relevant variance by obtaining 1000 bootstrapped atlas collections (of 35 atlases each) for the first 10 control subjects in our ADNI dataset and tracking for each subject the bootstrap variance of hippocampal volume estimates across increasingly larger numbers of bootstrapped atlas collections (ranging from 10 to 1000). As shown in Figure 2, the bootstrap variances for the 10 subjects stabilized at 300 (or fewer) bootstrapped atlas collections.

### 3.3 MRI Quality Metrics

To compare our precision weights to measures of image quality, we applied MRIQC (Esteban et al. 2017) to the unprocessed versions (neck‐trimmed only) of the 400 ADNI images used in our first experiment (200 from controls and 200 from AD subjects). The software's automated pipeline extracts more than 60 image quality metrics for each image, including noise‐based measurements, such as the signal‐to‐noise ratio (SNR), information theory‐based measurements, such as the foreground‐to‐background energy ratio (FBER, Shehzad et al. 2015), and artifact‐detecting measurements, such as Mortamet et al. (2009)'s quality indices (QI_1_ and QI_2_).

### 3.4 Precision Weights: Statistical Analysis

Knowing the precision of estimates enables calibrating error or bias in a variety of statistical analyses. For example, as described in Section 3.1, weighting each response by its precision maximizes efficiency in WLS. We will demonstrate the utility of our precision weights in the context of WLS because we invoked WLS theory to develop them and because WLS represents a common and effective method for understanding associations between brain volumetric data and biological or functional covariates.

In contrast to classical weighted regression settings where weights encode pre‐defined sampling probabilities, testing for associations using estimated weights requires carefully designed permutation‐based inference procedures to ensure scientific rigor. To derive a p‐value for the effect of disease status in WLS in the presence of nuisance covariates (in this case, age and ICV), we performed the weighted version of the Collins–Dekker permutation procedure (Dekker, Krackhardt, and Snijders 2007) for linear models, as described in ter Braak (2022). ter Braak (2022), which compared weight‐adjusted implementations of popular permutation methods, including the Freedman–Lane procedure (Freedman and Lane 1983), demonstrated that this method retains desirable power while controlling the type I error rate.

Specifically, we extracted the residuals from WLS regressing the predictor of interest against the nuisance covariates and generated new predictor vectors by permuting these residuals. For each permutation, we recorded the t‐statistic from WLS regressing the response against all the predictors (permuted residuals and nuisance covariates). We computed the proportion of the permutation‐derived t‐statistics and the t‐statistic from WLS on the unpermuted data (1000 t‐statistics altogether) with an absolute value exceeding the absolute value of the latter. Note that this procedure reduces to the standard Collins–Dekker method (referred to as the Smith method in Winkler et al. (2014)) when all the subjects share the same weight. (We also implemented the weighted Freedman–Lane procedure described in ter Braak (2022), but we omitted its results from Sections 4.1 and 4.2 for conciseness because it performed similarly.)

### 3.5 Precision Weights: Validation

We validated our precision weights by assessing how incorporating them affects the type I error rate and power in detecting a disease status effect on hippocampal volume in different subgroups of our ADNI data. In all of our experiments, we regressed the subjects' hippocampal volume estimates (obtained from JLF on the 35 OASIS atlases) against their disease status (CN vs. AD or amyloid‐negative CN vs. amyloid‐positive EMCI), ages, and intracranial volumes (ICVs). We included age and ICV as covariates, as recommended by Sanfilipo et al. (2004) and Barnes et al. (2010), to control for brain volume changes that accompany normal aging and the association between larger head sizes and larger brain volumes.

#### 3.5.1 Type I Error Rate

For each sample size n (ranging from 5 to 80), we sampled without replacement (to prevent overlap between our comparison groups) 2n of the 200 ADNI images from control subjects. We randomly assigned a group label to each subject (0 or 1) and performed WLS using the precision weights and identical weights of 1s (equivalent to OLS). We recorded the p‐value for the effect of our artificial group labels for both regressions and repeated this procedure 1000 times. We computed the proportions of p‐values <0.05 for the weighted and unweighted tests to compare their type I error rates.

#### 3.5.2 Power

In our first power experiment, for each sample size n (ranging from 5 to 160), we sampled with replacement n of the 200 ADNI images from control subjects and n of the 200 ADNI images from AD subjects and performed WLS using (1) our precision weights, (2) inverse coefficients of variation (CV), and (3) identical weights of 1 s (equivalent to OLS). We computed the CV for each subject's hippocampal volume estimate by dividing the standard deviation of the 300 bootstrapped hippocampal volume estimates by their mean. Although this ratio does not coincide with our bootstrap variance, it quantifies similarly the variability in each volume estimate. We included inverse CV‐weighting as an additional comparison (in addition to the baseline unweighted regression) because Roy et al. (2018) demonstrated its effectiveness in detecting associations between AD biomarkers and disease diagnosis. We recorded the p‐values for all three regressions (precision weights, inverse CV weights, and identity weights) and repeated this procedure 1000 times. We computed the proportions of p‐values <0.05 for the weighted and unweighted tests to compare their power.

In our second experiment, we conducted an analogous analysis with our ADNI images from 200 amyloid‐negative controls (i.e., without preclinical AD) and 200 amyloid‐positive EMCI subjects (i.e., with prodromal AD). Methods that effectively differentiate the latter from the former despite more subtle brain atrophy would facilitate disentangling prodromal AD and other causes of cognitive impairment and devising protocols for early detection and management to slow disease progression.

## 4 Results

**FIGURE 3:**
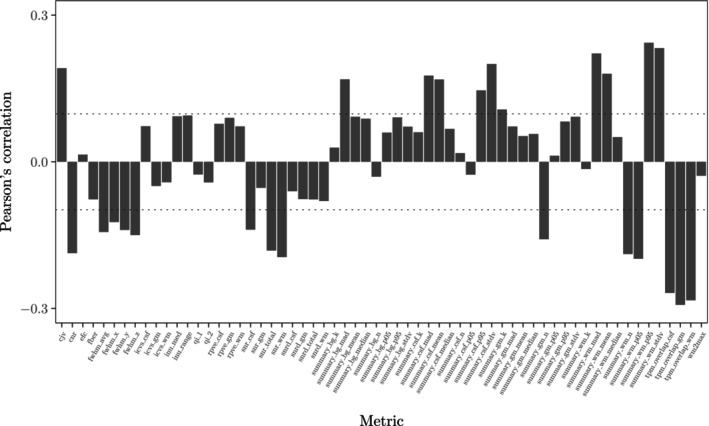
Each bar indicates the Pearson's correlation between an MRIQC metric and the bootstrap variance in the subset of our 400 ADNI images (200 AD subjects and 200 controls). The dotted lines demarcate the correlations that attain significance in the corresponding sample size.

The 62 MRIQC metrics that we evaluated include those that quantify noise and the presence of artifacts (including motion artifacts). As shown in Figure 3, the MRIQC metrics correlate weakly with the bootstrap variances, yielding Pearson's correlation coefficients ranging from −0.29 to 0.24. We obtained similar results (not shown) when we computed these correlations within each ADNI phase to accommodate the sensitivity of MRIQC to protocol differences (Garcia‐Dias et al. 2020). The statistically significant but low correlations for nearly half (25) of the MRIQC metrics suggest that our precision weights convey image quality but also encode additional information.

**FIGURE 4:**
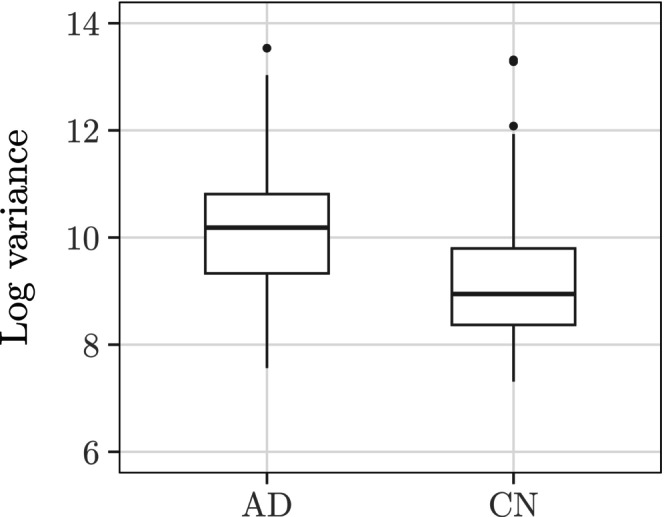
The boxplots summarize the distributions of the bootstrap variances of hippocampal volume estimates (in mm^3^) among 200 controls and 200 AD subjects in the ADNI. We plot the log variances to emphasize the bulk distributions. In general, the bootstrap variances for the volume estimates for the AD subjects exceeded the bootstrap variances for the volume estimates for the controls (p‐value <0.01 for a one‐sided t‐test comparing the mean log bootstrap variances in the two groups).

**FIGURE 5:**
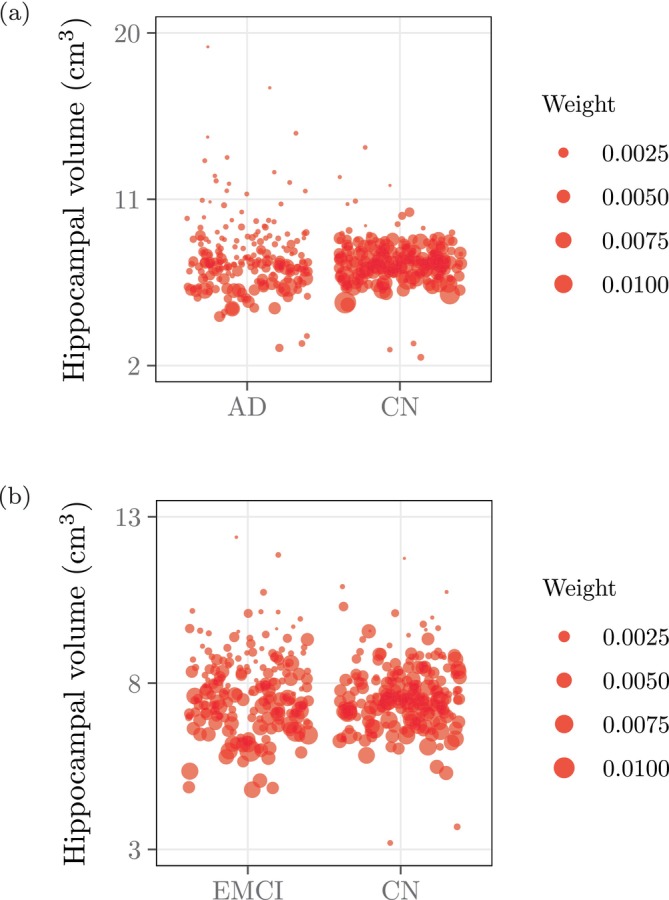
The scatterplots summarize the distributions of hippocampal volume estimates among different subgroups from the ADNI. The size of each point indicates the precision weight of the corresponding volume estimate in WLS. (a) Controls vs. AD subjects. (b) Amyloid‐negative controls vs. amyloid‐positive EMCI subjects.

Indeed, the uncertainty incurred during registration and segmentation depends also on interactions between the raw image and the processing pipeline. Figure 4 indicates that among our 400 ADNI images from controls and AD subjects, the images from the latter group generally produced more variable hippocampal volume estimates across bootstrapped atlas collections and therefore lower precision weights. Figure 5, which couples hippocampal volume estimates with their precision weights across different ADNI subgroups, underscores this observation by contrasting the disparity between the weights for the controls versus AD subjects (Figure 5a) with the similarity between the weights for the amyloid‐negative controls vs. amyloid‐positive EMCI subjects.

This difference signifies that our precision weights detect segmentation uncertainty due to alignment between the subjects contributing to the target images and the subjects contributing to the atlas images (healthy controls for the 35 OASIS atlases). Moreover, it supports previous findings that the choice of atlases impacts segmentation accuracy, especially when the target images exhibit disease‐related morphological changes (Carmichael et al. 2005; Yaakub et al. 2020). Thus, while convenient and common, using atlases from healthy controls to segment images from disease cohorts can undermine the impact of these images in downstream analyses. Matching atlases by covariates such as age or case–control status would mitigate this problem. More generally, using personalized brain maps, including personalized brain parcellations such as those developed by Salehi et al. (2017), would further diminish the uncertainty in our estimates.

### 4.1 Type I Error Rate

**FIGURE 6:**
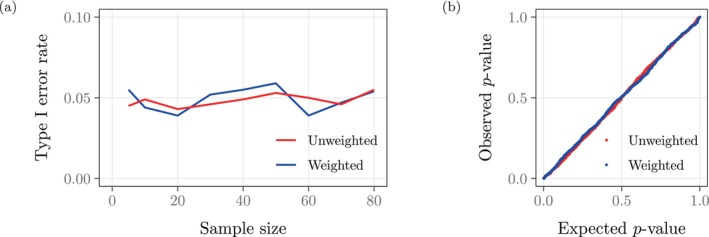
(a) For each sample size n, we sampled without replacement 2n images from control subjects and estimated, with and without precision weights, the effect of disease status on hippocampal volume while controlling for age and ICV. We recorded the p‐values for both regressions and repeated this procedure 1000 times. Each data point indicates the proportion of these 1000 p‐values <0.05. The weighted and unweighted methods both control the type I error rate. (b) We sampled without replacement 160 images from control subjects and estimated, with and without precision weights, the effect of disease status on hippocampal volume while controlling for age and ICV. We repeated this procedure 1000 times and constructed Q–Q plots for both sets of p‐values. As expected under the null hypothesis, the Q–Q plots nearly coincide with the y=x line; that is, the p‐value distributions from the weighted and unweighted methods both approximate the uniform distribution.

As shown in Figure 6a, our proposed weighting and permutation procedure controls the type I error rate across different sample sizes and incurs similar type I error rates as OLS, which ignores segmentation precision. In addition, Figure 6b displays the permutation p‐values from our null experiment on n=80 samples (see Section 3.5.1). The consistent type I error rates in Figure 6a and the alignment between the empirical and expected (uniform) p‐value distributions in Figure 6b also indicate the validity of the weighted version of the Collins–Dekker procedure for deriving p‐values semi‐parametrically.

### 4.2 Power

**FIGURE 7:**
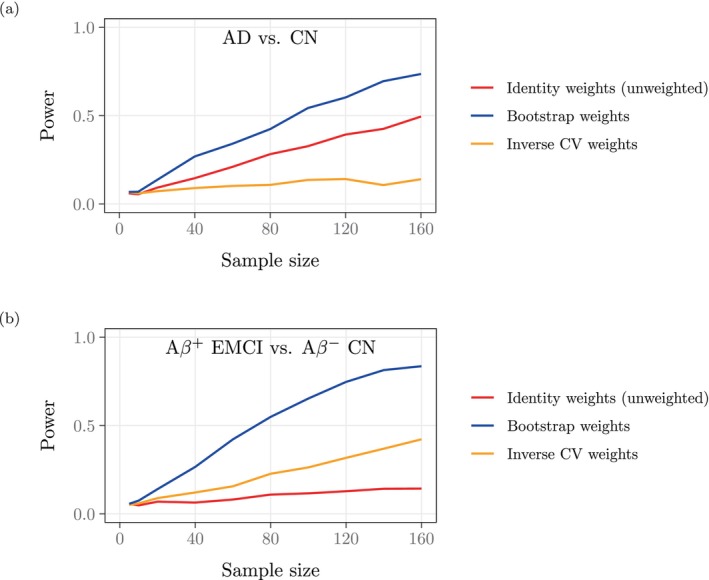
For each sample size n, we sampled with replacement n images from each group and estimated the effect of disease status on hippocampal volume while controlling for age and ICV. We conducted each regression with identity weights (unweighted), inverse CV weights, or our bootstrapping‐derived precision weights, recorded the p‐values for all three weighting schemes, and repeated this procedure 1000 times. Each data point indicates the proportion of these 1000 p‐values <0.05. Weighting increases the power dramatically. (a) Controls vs. AD subjects. (b) Amyloid‐negative controls vs. amyloid‐positive EMCI subjects.

Figure 7 indicates that weighting by our precision weights confers significantly increased power compared to OLS. For example, for n=160 samples, weighting improves the power to detect a difference in mean hippocampal volume between controls and AD subjects from 0.495 to 0.736 (Figure 7a). The power gain for differentiating between amyloid‐negative controls and amyloid‐positive EMCI subjects is even more impressive. For example, for n=160 samples, weighting improves the power from 0.143 to 0.836 (Figure 7b). Inspecting Figure 5 yields a possible explanation for the dramatic effect of weighting in our second experiment. Note that both the control group in the first experiment and the amyloid‐negative control group in the second experiment contributed both high and low outlying volume estimates, which received low precision weights, while the AD group in the first experiment and especially the amyloid‐positive EMCI group in the second experiment contributed mostly high outlying volume estimates (which also received low precision weights). We hypothesize that in the second experiment, the larger precision weights for the lower volume estimates among the EMCI subjects (Figure 5b) afforded the advantage necessary to differentiate the two groups, which are otherwise indistinguishable by hippocampal volume, whereas in the first experiment, the already‐substantial volume differences between controls and AD subjects restricted the potential improvement conferred by weighting.

Moreover, note that in the first experiment, the unweighted analysis outperformed inverse CV‐weighting (0.140 power at n=160), while in the second experiment, inverse CV‐weighting (0.422 power at n=160) outperformed the unweighted analysis. The leading performance of our precision weights in both experiments suggests that although CV weights share similar motivations as our bootstrapping‐derived precision weights, this proxy for uncertainty cannot guarantee similarly consistent power gains as our theory‐driven approach. These quantitative results, combined with our observation of low precision weights computed for outliers, most evident in the high volume estimates in Figure 5a, establish the downstream impact of our method's discernment in correctly downweighting these values.

## 5 Discussion

In this paper, we have proposed a novel way of deriving subject‐level weights that quantify the variability in volume estimates across joint segmentations produced by different atlas collections. We have also demonstrated on real data that incorporating these weights significantly improves power for detecting a mean group difference in hippocampal volume. These results encourage a transition in approaching imaging studies. Just as image quality metrics (Esteban et al. 2017) formalized systematic ways of curating imaging data, our segmentation precision metric and weighted regression framework establish a promising tool for optimizing imaging analyses.

Our method is easy to understand and straightforward to implement while still recognizing that each subject's ROI volume estimate depends on both the quality of the raw image and its behavior during segmentation, which are related but non‐identical. The former comprises artifacts incurred during image acquisition while the latter indicates compatibility with atlases due to alignment in covariates (such as age or disease status) or brain morphology (such as sulcal patterns). Intuitively, bootstrapping on atlases to estimate variability resembles proposed techniques for calibrating uncertainty in deep learning‐based segmentation methods, including varying dropout layers, input labels, model ensembles, loss functions, and other features (Abdar et al. 2021; Zou et al. 2023). However, with the exception of Roy et al. (2018), these works have primarily evaluated their uncertainty measures via segmentation or predictive accuracy. In contrast, our method outputs precision weights that confer substantial improvements in downstream statistical analyses. Thus, it provides definite guidance on how to leverage the uncertainty information extracted from the segmentation procedure to facilitate testing hypotheses motivated by biological questions.

We envision several directions for generalizing and applying our method. For example, we chose JLF for label fusion in all of our experiments, but our bootstrapping procedure transfers easily to other MALF strategies, as highlighted in Figure 1, or segmentation methods that involve aggregating multiple inputs. For example, the deep learning‐based segmentation method nnU‐Net (Isensee et al. 2021), which remains a state‐of‐the‐art framework for automated deep learning‐based segmentation, trains three different models and employs cross‐validation to determine an optimal model (or model ensemble) for each dataset. The parallel between different atlas collections and different model ensembles renders nnU‐Net an attractive candidate for adapting our precision weights. Furthermore, the strategy that we described in Section 3.2 to determine the number of bootstrap replicates pertains to all such applications.

Moreover, in addition to volumetric studies, we can compute analogous weights for any type of analysis that utilizes information from segmented ROIs, such as ROI‐level cortical thickness, fractional anisotropy, or even functional signal. In fact, for secondary measures that can differ substantially across contiguous regions, our precision weights might benefit analyses even more because such measures exhibit greater sensitivity to segmentation accuracy.

In this paper, we have estimated subject‐level variances of hippocampal volume estimates based on across‐atlas set variation. However, the whole brain segmentations that we obtained via JLF to extract these volume estimates provide additional information that we neglected in our experiments. We emphasize that for each subject, the joint segmentations from bootstrapped atlas collections enable estimating simultaneously this variance for any ROI. In fact, they generate a subject‐level approximation of the covariance structure between volume estimates of different ROIs. Thus, without requiring additional computational resources, we can extend our method to conduct mass univariate weighted regressions against ROI volume estimates or weighting‐informed multivariate analyses comparing subject‐level vectors of ROI volume estimates. These analyses can potentially reveal new information about regional brain changes during disease progression by remedying the problem that some disease‐informative ROIs are inherently more difficult to segment.

Finally, we anticipate that the subject‐level uncertainty measures of imaging‐derived estimates would also facilitate other analyses that arise in clinically relevant settings. For example, extensive interest prevails in predicting phenotypes from biomarkers, including imaging‐derived features. Probing such questions often entails regressing a scalar functional measure on multiple biomarkers and predicting measures for new subjects from the derived effect sizes. This application differs from our experiments in that our precision weights now indicate the variability in the independent variables. For such analyses, they could function as correction factors to calibrate the attenuation bias in our predictions (Carroll, Ruppert, and Stefanski 1995) and thus refine the utility of imaging biomarkers in monitoring disease progression or treatment effects.

In terms of limitations, our method estimates the *conditional* variance of each subject's ROI volume estimate given the corresponding target image but ignores the expected variability among multiple scans of the same subject acquired under identical conditions. Resolving this gap appears fundamentally difficult in the absence of scan‐rescan data. Much work remains in formalizing how to integrate these two sources of uncertainty to derive subject‐specific metrics and determining how much additional power doing so will contribute beyond what our precision weights already achieve.

## Conflicts of Interest

DAW has served as a paid consultant to Qynapse, Beckman Coulter, and Eli Lilly. He serves on a DSMB for GSK. He also received grants from the NIH and Biogen paid to his institution and travel support from the Alzheimer's Association. RTS has received consulting income from Octave Bioscience and compensation for scientific reviewing from the American Medical Association.

## Supporting information


Data S1.

## Data Availability

The data that support the findings of this study are openly available in LONI Image Data Archive at http://adni.loni.usc.edu.
