# Supplementary material for: Subject‐Level Segmentation Precision Weights for Volumetric Studies Involving Label Fusion
Source: Hum Brain Mapp. 2024 Dec 19;45(18):e70082. doi: 10.1002/hbm.70082 (PMC11656102; doi:10.1002/hbm.70082)
Supplement: Supplementary file 1 — Data S1. [file HBM-45-e70082-s001.pdf]

Retain the setup from the main document.

Proposition 1. *Consider the WLS estimator*

$$\hat{\boldsymbol{\beta}} \equiv (\boldsymbol{X}^t\boldsymbol{\Sigma}^{-1}\boldsymbol{X})^{-1}\boldsymbol{X}^t\boldsymbol{\Sigma}^{-1}\boldsymbol{Y}$$

obtained by inverse variance-weighting. If $\tilde{\boldsymbol{\beta}} \equiv \boldsymbol{P}\boldsymbol{Y}$ is any other unbiased linear estimator of $\boldsymbol{\beta}$, then

$$\mathrm{Var}[\tilde{\boldsymbol{\beta}} \,|\, T_1, \ldots, T_n, \boldsymbol{X}] - \mathrm{Var}[\hat{\boldsymbol{\beta}} \,|\, T_1, \ldots, T_n, \boldsymbol{X}] \tag{1}$$

is positive semidefinite.

Proof. Consider any linear estimator $\tilde{\boldsymbol{\beta}} \equiv \boldsymbol{P}\boldsymbol{Y}$ of $\boldsymbol{\beta}$ and define

$$\boldsymbol{D} \equiv \boldsymbol{P} - (\boldsymbol{X}^t\boldsymbol{\Sigma}^{-1}\boldsymbol{X})^{-1}\boldsymbol{X}^t\boldsymbol{\Sigma}^{-1}.$$

Note that

$$\mathbb{E}[\tilde{\boldsymbol{\beta}} \,|\, T_1, \ldots, T_n, \boldsymbol{X}] = [\boldsymbol{D} + (\boldsymbol{X}^t\boldsymbol{\Sigma}^{-1}\boldsymbol{X})^{-1}\boldsymbol{X}^t\boldsymbol{\Sigma}^{-1}]\boldsymbol{X}\boldsymbol{\beta} = (\boldsymbol{D}\boldsymbol{X} + \boldsymbol{I}_p)\boldsymbol{\beta},$$

where $\boldsymbol{I}_p$ indicates the $p \times p$ identity matrix, so $\tilde{\boldsymbol{\beta}}$ is unbiased iff $\boldsymbol{D}\boldsymbol{X} = \boldsymbol{0}_{p\times p}$, in which case

$$\begin{aligned}
\mathrm{Var}[\tilde{\boldsymbol{\beta}} \,|\, T_1, \ldots, T_n, \boldsymbol{X}] &= [\boldsymbol{D} + (\boldsymbol{X}^t\boldsymbol{\Sigma}^{-1}\boldsymbol{X})^{-1}\boldsymbol{X}^t\boldsymbol{\Sigma}^{-1}]\boldsymbol{\Sigma}[\boldsymbol{D}^t + \boldsymbol{\Sigma}^{-1}\boldsymbol{X}(\boldsymbol{X}^t\boldsymbol{\Sigma}^{-1}\boldsymbol{X})^{-1}] \\
&= \boldsymbol{D}\boldsymbol{\Sigma}\boldsymbol{D}^t + \underbrace{\boldsymbol{D}\boldsymbol{X}(\boldsymbol{X}^t\boldsymbol{\Sigma}^{-1}\boldsymbol{X})^{-1}}_{\boldsymbol{0}} + \underbrace{(\boldsymbol{X}^t\boldsymbol{\Sigma}^{-1}\boldsymbol{X})^{-1}\boldsymbol{X}^t\boldsymbol{D}^t}_{\boldsymbol{0}} + (\boldsymbol{X}^t\boldsymbol{\Sigma}^{-1}\boldsymbol{X})^{-1} \\
&= \boldsymbol{D}\boldsymbol{\Sigma}\boldsymbol{D}^t + \mathrm{Var}[\hat{\boldsymbol{\beta}} \,|\, T_1, \ldots, T_n, \boldsymbol{X}].
\end{aligned}$$

Thus, (1) equals $\boldsymbol{D}\boldsymbol{\Sigma}\boldsymbol{D}^t$ and is indeed positive semidefinite. □

Note that the positive semidefinite property in Proposition 1 is equivalent to

$$\mathrm{Var}[\boldsymbol{\ell}^t\tilde{\boldsymbol{\beta}} \,|\, T_1, \ldots, T_n, \boldsymbol{X}] \geq \mathrm{Var}[\boldsymbol{\ell}^t\hat{\boldsymbol{\beta}} \,|\, T_1, \ldots, T_n, \boldsymbol{X}] \ \forall\, \boldsymbol{\ell} \in \mathbb{R}^p.$$

In particular, if the first covariate in $\boldsymbol{X}_i$ encodes the disease group of the i-th subject, then taking $\boldsymbol{\ell}$ to be the first standard basis vector yields

$$\mathrm{Var}[\tilde{\beta}_1 \,|\, T_1, \ldots, T_n, \boldsymbol{X}] \geq \mathrm{Var}[\hat{\beta}_1 \,|\, T_1, \ldots, T_n, \boldsymbol{X}].$$
